# Sex Differences in Risk Preference and c-Fos Expression in Paraventricular Thalamic Nucleus of Rats During Gambling Task

**DOI:** 10.3389/fnbeh.2018.00068

**Published:** 2018-04-10

**Authors:** Hironori Ishii, Mariko Onodera, Shinya Ohara, Ken-Ichiro Tsutsui, Toshio Iijima

**Affiliations:** ^1^Division of Systems Neuroscience, Tohoku University Graduate School of Life Sciences, Sendai, Japan; ^2^Department of Experimental Psychology, University of Oxford, Oxford, United Kingdom

**Keywords:** risk, decision making, rats, sex difference, paraventricular thalamic nucleus

## Abstract

Different biological requirements between males and females may cause sex differences in decision preference when choosing between taking a risk to get a higher gain or taking a lower but sure gain. Several studies have tested this assumption in rats, however the conclusion remains controversial because the previous real-world like gambling tasks contained a learning component to track a global payoff of probabilistic outcome in addition to risk preference. Therefore, we modified a simple gambling task allowing us to exclude such learning effect, and investigated the sex difference in risk preference of rats and its neural basis. The task required water deprived rats to choose between a risky option which provided four drops of water or no reward at a 50% random chance vs. a sure option which provided predictable amount *x* (*x* = 1, 2, 3, 4). The amount and the risk were explicitly instructed so that different choice conditions could be tested trial by trial without re-learning of reward contingency. Although both sexes correctly chose the sure option with the same level of accuracy when the sure option provided the best offer (*x* = 4), they exhibited different choice performances when two options had the same expected value (*x* = 2). Males and females both preferred to take risky choices than sure choices (risk seeking), but males were more risk seeking than females. Outcome-history analysis of their choice pattern revealed that females reduced their risk preference after losing risky choices, whereas males did not. Rather, as losses continued, reaction time for subsequent risky choices got shorter in males. Given that significant sex difference features mainly emerged after negative experiences, male and female rats may evaluate an unsuccessful outcome of their decision in different manners. Furthermore, c-Fos expression in the paraventricular nucleus of the thalamus (PV) was higher in the gambling task than for the control task in males while c-fos levels did not differ in females. The present study provides a clear evidence of sex differences in risk preference in rats and suggests that the PV is a candidate region contributing to sex differences in risky decision making.

## Introduction

Many animal species exhibit sex different characteristics not only in their body features but also in behavioral traits including foraging strategy (reviewed in Williams, 1957 and in Dienstbier et al., 2001; e.g., monkey: Boinski, 1988, elephant seal: Le Boeuf et al., 1993, giraffe: Ginnett and Demment, 1997, penguin: Clarke et al., 1998). Sex differences can also emerge in decision making where a decision maker is given a choice between an option which provides a smaller but guaranteed gain and an option which possibly provides a larger gain but also could provide a loss. When outcomes are uncertain, the outcome with known probability is defined as “risk” (the outcome with unknown probability is defined as “ambiguity”) in foraging studies and neuroeconomics (Stephens and Krebs, 1986; Glimcher et al., 2009; Burke and Tobler, 2011). Risk has impact on human’s and animal’s decision making even if an alternative with the risk has the same mathematical expected value/global payoff as another sure alternative (mathematically equivalence point). The choice preference on the mathematically equivalence point is called “risk preference”, and a decision maker is categorized as “risk seeking”, “risk neutral” or “risk aversive” based on this risk preference (Stephens and Krebs, 1986; Platt and Huettel, 2008; Glimcher et al., 2009). Note that term “risk preference” is distinguished from (reinforcement) learning process to track the changes in global payoff of probabilistic outcome in the framework of decision making. “Risk proneness” has been used to indicate when the risk is not only exploited, but also tracked along its variations in the probabilistic delivery task (Zoratto et al., 2016). In humans, it is well established that males tend to be more risk seeking than females in a wide domain of decision making (Weber et al., 2002), gambling (Raylu and Oei, 2002; van den Bos et al., 2013a,b) and financial risk taking (Powell and Ansic, 1997; Dwyer et al., 2002; Eckel and Grossman, 2002; Charness and Gneezy, 2012; Arora and Kumari, 2015).

In contrast, studies on non-human animals including common laboratory animals, rats and mice, have been limited in their conclusions about sex differences in risk preference. Therefore the behavioral and neural mechanisms of sex differences in risk preference remain unclear. One controversial point in the previous studies in rats is that complexities of behavioral tasks did not eliminate several alternative interpretations other than risk preference, which may account for inconsistent results. For example, male rats were less risk seeking than females in the rodent Iowa gambling task (rIGT) where subjects were required to develop choices for an option provided frequent small sugar pellets and occasional quinine which resulted in long term advantage over an option provided occasional large sugar food among frequent punishments of quinine (van den Bos et al., 2012). However, two contradictory results have been reported in a variant of rIGT which is a choice among several options with differing reward amounts, probability and duration of time-out penalty where a subject had to wait to start the next trial. In one study, no sex differences emerged (Peak et al., 2015) but in another study female rats were less risk seeking than males (Georgiou et al., 2018). Furthermore, female rats were less risk seeking than males when choosing between a larger food accompanied by a probabilistic foot-shock vs. a smaller food but with no shock (Orsini et al., 2016).

However, since these behavioral tasks were designed as animal models of neuropsychiatric disease or real-world like decision making, they are suitable for first screening for abnormality in decision making. They are ill-suited to evaluating sex differences in risk preference independently from other factors as mentioned below. One issue is that sex differences in sensitivities or tolerances to different punishment types (quinine, time-out, or foot-shock) may exist. The second issue is potential sex differences in learning. The rIGT is designed to assess learning about long term gain of probabilistic reward through multiple rewarded/unrewarded experiences. Thus, these studies of sex differences in risk preference (van den Bos et al., 2012; Peak et al., 2015; Georgiou et al., 2018) might reflect sex difference in learning (payoff detection) rather than risk preference. The learning issue also arises in the technical aspect of the other tasks. For instance, in another study of sex differences in risk preference (Orsini et al., 2016) the risky option was examined against different probabilities (0, 25, 50, 75, 100%) block by block with progressive order. This method required the rats to update/re-learn the new reward contingency block by block (payoff tracking). However, males and females show differences in rate of learning during initial acquisition, extinction, and reacquisition in Pavlovian and operant conditioning tasks (Dalla and Shors, 2009; Hammerslag and Gulley, 2014) and as suggested by the study mentioned initially (van den Bos et al., 2012).

Given these issues, we employed a simpler gambling task focusing on decision making based on reward amount and probability (Logan, 1965; Caraco et al., 1980; Ishii et al., 2012, 2015). The task was a choice between a risky option which provided four drops of water or no reward at a 50% random probability vs. a sure option which provided predictable reward amount *x* (*x* = 1, 2, 3, 4). Here, reward probability for a risky option is fixed and, instead, reward amount for a sure option is manipulated since learning reward probability generally needs a lot of experience so that manipulating probability is likely to accompany the second issue, influence of payoff detection and tracking. Indeed, in a manipulation of reward probability across 12.5, 25, 50, 100%, rats are unable to track the changes correctly when the manipulation was conducted with scattered order (St. Onge et al., 2010). A novel addition to the task to minimize any learning effects was that reward amount and its risk were explicitly instructed by number of LED and its illumination pattern (described in “Materials and Methods” section). This procedure enabled us to randomize test conditions trial by trial without the need for re-learning. This is also an important improvement to avoid the carry-over effects of previous test conditions. In contrast, other decision making tasks, such as the probabilistic discounting task (St. Onge et al., 2010) and the probabilistic delivery task (Zoratto et al., 2016), use block by block or session by session methods and progressive testing order in a manipulation of reward probability which might be affected by carry-over effects.

The main purpose of this task was to test the choice preference where the risky and sure options had the same expected value (*x* = 2) while the other three choice conditions (*x* = 1, 3, 4) were control conditions to prove that the rats were sensitive to relative value of the two options. In our previous studies, male rats showed a preference for the risky option in this choice condition (Ishii et al., 2012, 2015). Here, we tested for a difference in the risk preferences of male and female rats in Experiment 1 and focused more on their outcome-history based choice pattern in Experiment 2. In addition, in Experiment 3, we looked for a sex difference in c-Fos expression for the task. In this report, we focused on the paraventricular nucleus of the thalamus (PV) which is thought to be a key region that controls approaching/avoidance behavior switching (Hsu et al., 2014; Kirouac, 2015; Do-Monte et al., 2016; Choi and McNally, 2017). In particular, a recent study found that the PV control was recruited only in unpredictable reward omission (Do-Monte et al., 2017). Therefore, the PV is hypothesized to be prominent in win/lose-based gambling choices rather than simple stereotypic discrimination choices and is also hypothesized to reflect sex difference win/lose-based choice preference.

## Materials and Methods

### Animals and Housing

Eleven male and 13 female Wistar rats, 8 weeks of age, were first acclimatized to the housing environment and human handling for 1 week before they served in the experiment. Rats were individually housed under 12 h light/dark cycles with light onset at 8:00 PM. Behavioral training and testing took place during the dark phase because the rats are nocturnal. In the home cage, rats were given *ad libitum* access to food for the duration of the experiments but not given water because water was used as a reward in the experiment. To prevent weight loss, their body weights were monitored daily, and if necessary, they were given additional water after the daily experiment finished. For instance, if the rat needed 15 mL to keep his/her body weight and got 12 mL throughout the experiment, we provided additional 3 mL water at the end of day. The procedures under living animal in this project was licensed (2016LSA-010) by the Institutional Animal Care and Use Committee of Tohoku University and was conducted in accordance with the Guidelines for Animal Care and Use of Tohoku University.

### Apparatus

A dimly lit sound-attenuated box (60 × 45 × 35 cm) was divided into the behavioral task arena and device storage. The box was equipped with a ventilation fan. Gate open/close was controlled by micro-servo motor (SG92R, Tower Pro Pte Ltd.). Gate entry and nose-poke response were detected by infrared beam (RPR220, OMRON). White LEDs were positioned above the stainless nozzles. Water delivery was controlled by solenoid valve (JTV-2, TAKASAGO ELECTRIC, INC.). Auditory stimuli were generated by a speaker located in the device storage. Each device was connected to a computer via a digital I/O card (PCI-7248, ADLINK Technology) and controlled by an in-house software program (based on Visual C++ MFC application, Visual studio 2015).

### Gambling Task

**Figure 1:**
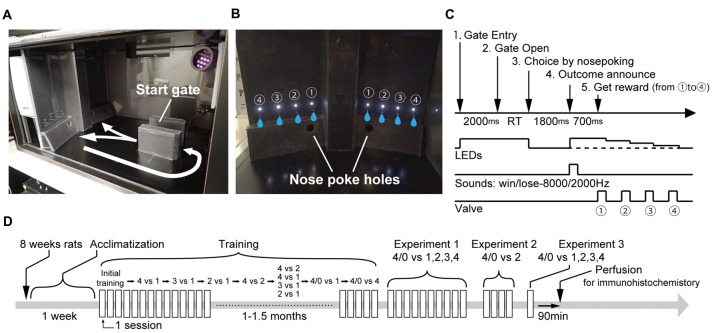
Task design. **(A,B)** Experimental apparatus. Number of illuminating LED cues indicated the reward amount. Flashing/continuous illumination of LED cues indicated risky/sure. **(C)** Sequence of events in a trial. Rats voluntarily initiated a trial by entering the start gate and waiting for 2 s until the gate opened. The offers of the two panels were presented after entry. The rats made a choice by nose-poking to either the right or left nose-poke hole. After a delay period (1800 ms), a sound announcing rewarded/non-rewarded was given before reward delivery. The reward was provided sequentially from ① to ④ 1 s apart. **(D)** Experimental schedule.

The basic concept of the task was the same as what we previously used (Ishii et al., 2012, 2015) but was improved in several ways to produce more stable and reliable behavioral performance. The risky option offered an unpredictable outcome which resulted in four drops of water or no reward at a random chance of 50%. The sure option offered predictable outcomes of 1, 2, 3, or 4 drops randomly selected in each trial. The offered reward risk and amount were explicitly instructed by LED illumination at the beginning of each trial. Two options were assigned to the right and left panels (Figure 1). Each panel was equipped with four pairs of a nozzle and LED. Each nozzle provided a 50 μl water drop. The LED cue indicated whether the paired nozzle provided a reward or not. That is, the number of illuminating LED cues indicated the reward amounts. In addition, flashing/continuous illumination of the LED cue indicated risky/sure. The assignment was counter balanced between subjects. They could easily distinguish the number of illuminating LEDs or flashing/continuous conditions. However, they did not show any choice preference regarding the LED brightness or flashing themselves. The reward was provided sequentially from the medial to lateral side nozzle, 1 s apart.

### Trial Structure

The trial structure is shown in Figure 1. A trial began by the rats voluntarily entering the start gate. Then, two offers were presented on each panel. To let them know each offer, the rats were required to wait for 2 s until the gate was opened. If the rats escaped from start gate by going back before the gate opened, the trial was canceled and they were required to redo it from the beginning. However, this situation rarely happened. After the gate opened, the rats made a choice by poking their nose through the right or left nose-poke hole. At this timing, all the LED cues were turned off (the LED cues on the unchosen side panel were also turned off). One-thousand and eight-hundred millisecond after making the choice, the outcome was announced by the LED cues and supportive auditory instructions. In a rewarded trial, LED cues on chosen side re-lighted and a sound indicating the reward was given. Reward delivery started 700 ms after the announcement, and the LED cues were turned off after the paired nozzle provided the reward. In a non-rewarded trial, the LEDs did not re-light and only a sound indicating no reward was given. The sounds were 8000 Hz and 2000 Hz and the assignment was counter balanced between subjects.

### Behavioral Training Procedures

On the first day, the rats were acclimatized to the task box during 30 min and water deprivation started form the night. From the second day, behavioral training was conducted with initial three steps. The first step taught them that poking their noses into either of two holes provided water from the nozzle (free choice between 4 drops vs. 4 drops without the entry to the start gate). In the second step, the rats were trained to nose-poke into the hole specified by illumination of the LED cue (forced choice; only one side of the LED cues was presented: the reward amount was fixed at four drops). In the third step, the rats were trained to enter the start gate to illuminate the LED cues. The trial type was still a forced choice. The reward amount changed trial by trial in a range of 1–4 drops.

After they had been trained with the basic rules of a trial, the rats were trained in the discrimination of the reward amount choices. First, they were trained in the free choice of 4 drops vs. 1 drop. Location assignments of the two options were changed at random from one trial to the next. Each session took 45 min. Sessions were conducted up to three times a day and were conducted more than 3 h apart. The rats were moved to the next discrimination choices when they had performed three consecutive sessions in which they chose the better option more than 80% of the time. Most of the rats passed this criterion within one or 2 days. Then, the rats were trained in the choice of 3 drops vs. 1 drop, 2 drops vs. 1 drop, and 4 drops vs. 2 drops one by one. Finally, the rats were trained in the mix of above-mentioned choice conditions. The criterion to finish this step was over 80% correct for each choice condition. The rats came to be able to perform the task with over 90% correctness in most sessions.

After the rats were trained to discriminate the reward amount, the risky option was introduced. The rats were exposed to the choice of 4/0 drops vs. 1 drop and 4/0 drops vs. 4 drops. These two choice patterns were alternately tested in different sessions in the first six sessions and were tested in the same session in the next three sessions. After the experience of the risky option, the rats were moved to Experiment 1. The whole training took from one month to one and half months.

### Experiment 1

**Figure 2:**
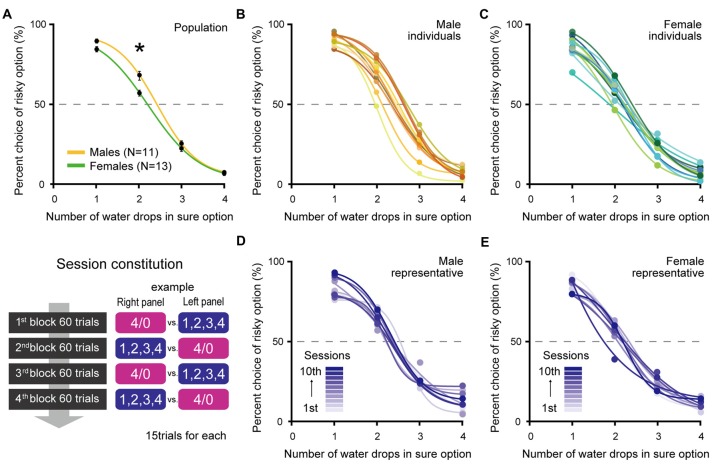
Percent choices of the risky option at population level **(A)** of males (*N* = 11) and females (*N* = 13), at individual level **(B,C)** and at session level of representative individuals **(D,E)** in Experiment 1. **(A)** Both males and females significantly preferred the risky option when *x* = 2 compared to chance level (both *p* ≪ 0.01). However, males were more risk seeking than females (*p* ≪ 0.01, indicated by asterisk). Error bars indicate SEM. **(B,C)** Individual differences in risk preference also emerged. Each color indicates the choice performance of each individual. Risky choices when *x* = 2 were significantly more frequent than chance level in 10 males and 8 females, but were not significant in one male and five females. **(D,E)** The choice performances within individuals were stable and did not progressively shift among the 10 sessions. Each color indicates the choice performance in each session.

Experiment 1 tested the performances in the choices of 4/0 vs. 1, 4/0 vs. 2, 4/0 vs. 3 and 4/0 vs. 4. Since the rats have preferences for spatial factors to some extent (e.g., preferring the right side or a clockwise turn), the locations of the risky and sure options were switched within a session to cancel out this effect. A session consisted of 240 trials divided into four blocks (60 trials for each). In a block, the locations of the risky and sure options were fixed but the choice conditions were changed trial by trial at random (15 trials for each). The location assignment was switched in the next block. The location assignment of the first block was counter balanced between sessions. A session took about 50 min on average. More than 10 sessions were conducted, and the last 10 were used for the individual data. A statistical analysis was conducted using R (R studio, version 3.3.2) and Microsoft Excel. The choice rate was expressed as the percentage of risky option choices. The curves of the risky choice changes in Figures 1, 2 were calculated by fitting a logistic sigmoid function (*f*(*x*) = *a* + *b*/(1 + exp (− (*x* − *c*)/*d*); *a*, *b*, *c* and *d* were free parameters) to the observed choice rates by using the least-squares method. Comparisons between percentage of the risky option choices and chance level (50%, random choice between the two options) were made using a one-sample *t-test* (significance level: *p* < 0.05). Comparisons between males and females were made using Welch’s *t*-test (significance level: *p* < 0.05). In addition, to check the effect of sex difference in body weight, correlations between the percentage choice of the risky option in the choice of 4/0 vs. 2 and body weight were tested using the Pearson product-moment correlation coefficient (significance level: *p* < 0.05).

### Experiment 2

**Figure 3:**
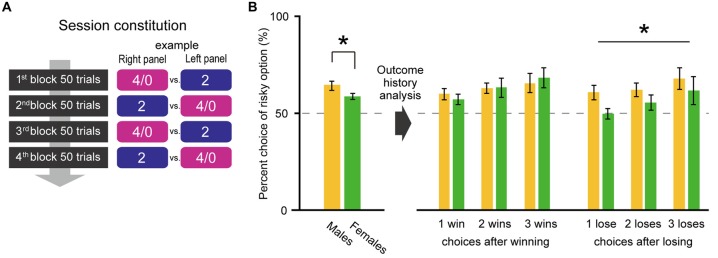
To analyze the choice history, only the 4/0 drops vs. 2 drops choice condition was tested in Experiment 2 **(A)**. Male rats exhibited higher risk preference than females again (*p* = 0.04; **B** left). However, the choice history analysis revealed that the sex difference occurred in the trials after a losing gamble in the previous trial (**B** right). The percent choices of risky option between two sexes were significantly different after losings (Sex: *p* = 0.04 by two-way ANOVA [Sex × Number of lose]), especially after one lose (*t*_(19)_ = 2.49, *p* = 0.02 by Welch’s* t*-test), whereas those were not after winnings (Sex: *p* = 0.98 by two-way ANOVA [Sex × Number of win]). The significant *p* values are indicated by asterisks. Error bars indicate SEM.

To analyze the outcome-history-based choice pattern, Experiment 2 tested the performance only in the case of the 4/0 vs. 2 choice (Figure 3A). A session consisted of 200 trials, and that was divided into four blocks (50 trials for each). Other procedures were the same as Experiment 1. A session took about 40 min on average. More than four sessions were conducted, and the last four were used for the individual data. For the choice history analysis, the trials were sorted according to the choice and outcome of the previous trial. Here, getting four drops as a result of making a risky choice is called “win” and getting no reward is called “lose”. “After winning (losing)” means the performance in a trial of which the previous trial was a rewarded (not rewarded) risky choice. The rest was the performance in a trial of which the previous trial was a sure choice. Comparisons between after winning and after losing were made using a paired *t*-test. All significance levels were *p* < 0.05. Other comparisons were conducted with the same way as Experiment 1.

### Experiment 3

To test c-Fos expression for the task, finally the rats were exposed to a single session of the gambling task and were sacrificed 90 min after the behavioral test. The task was the same as the single session of Experiment 1. The control rats were exposed to reward amount discrimination choices of 4 drops vs. 2 drops for 50 min, which is the average time to finish the gambling task. Six males and six females served in the gambling task, and four males and three females served in the control task.

The rats were anesthetized with an overdose of isoflurane and were transcardially perfused with saline and 4% PFA in 0.1 M phosphate buffer (PB). The brain samples were post-fixed in the same fixative solution for 24 h at 4°C and were cryoprotected for at least 48 h at 4°C in PB containing 30% sucrose before sectioning. The brain samples were coronally sectioned at 40 μm by using a freezing microtome. In order to identify the activated neurons, free floating sections were immunohistochemically processed for c-Fos and counterstained for the neuronal marker NeuN. The sections were first washed with phosphate-buffered saline (PBS) and soaked in PBS containing 5% goat serum and 0.1% Triton X-100 (Blocking solution) for an hour at room temperature. The sections were then incubated overnight at 4°C with rabbit polyclonal anti-c-Fos antibody (1:1000; Santa Cruz Biotechnology, sc-52) and mouse polyclonal anti-NeuN antibody (1:1000; Millipore, MAB377) dissolved in the same blocking solution for the primary antibody reaction. Sections were subsequently washed and permeabilized three times in PBS containing 0.1% Triton X-100 (PBT) and incubated for 4 h at room temperature in Cy3-conjugated goat anti-rabbit IgG (1:400; Jackson ImmunoResearch, 111-165-144) and Alexa488-conjugated goat anti-mouse IgG (1:400; Jackson ImmunoResearch, 115-545-146) diluted in PBT for the secondary antibody reaction. After washing three times with PBS, the sections were mounted on gelatinized slides. The well dried sample slides were dehydrated with xylene and were coverslipped with mounting medium (Entellan New, Merck Millipore, Burlington, MA, USA).

The sections were examined under a Zeiss Axiovert 200 M microscope and imaged using an AxioCam MRm digital camera (Carl Zeiss) and Axiovision image processing software (Carl Zeiss). In the present study, we focused on the paraventricular nucleus of the thalamus. Boundaries of anatomical regions were determined by using the rat brain atlas of Paxinos and Watson (2007). Brain regions were counted on sections 160 μm apart, four to five slices for each animal. Counts were averaged for each structure of interest in each animal. Counting was conducted by persons who were blind to the behavioral experiment. The number of c-Fos positive cells in the PV were compared between four groups (sex [male/female] × task type [gambling task/control task]) by using a two-way ANOVA and *post hoc* simple main effect test (significance level: *p* < 0.05).

## Results

### Experiment 1

Figure 2A shows the choice performance of male rats (*N* = 11) and female rats (*N* = 13) in terms of population level. First of all, the most important point is that all male and female rats properly chose the sure option when four drops was the best offer in this task. No sex difference emerged in this choice condition (*t*_(22)_ = 0.58, *p* = 0.57 by Welch’s *t*-test) indicates that both males and females performed this task with the same level of accuracy. However, the choice rates of males and females diverged as the value of the sure option became lower. When *x* was 2, although both males and females showed significant preference for the risky option compared with the chance level (males: *t*_(10)_ = 6.23, *p* ≪ 0.01, females: *t*_(12)_ = 4.04, *p* < 0.01 by one sample *t*-test), males were much more risk seeking than females (*t*_(17)_ = 3.22, *p* < 0.01 by Welch’s *t*-test). When *x* was 1, the gap between the two sexes got narrower but was still significant (*t*_(20)_ = 2.46, *p* = 0.02 by Welch’s *t*-test).

In addition to a sex difference, individual differences also emerged in risk preference. Figures 2B,C show the individual choice performances. On the basis of the choice rate when *x* was 2, 10 out of 11 male rats were risk seeking and one was risk neutral (by one sample *t*-test against chance level). In the females, 8 out of 13 rats were risk seeking and five rats were risk neutral.

Figures 2D,E show representative examples of choice performances in 10 sessions within the individuals. Choice performances were stable between sessions; the averages of the standard deviation of choice rate when *x* was 2 over the 10 sessions were 7.2 in males and 8.3 in females. Choice performances were also stable between blocks; no significant effect of blocks was detected by the repeated measures two-way ANOVA (Choice conditions × Blocks; Choice condition: *p* < 0.01, Blocks: *p* = 0.99, interaction: *p* = 1, for both sex). There was a possibility that rats got satisfied with water as they digested trials and changed their risk preference in the later block. However, in the present procedure, we need not take into account such an effect.

Although the choice rates when *x* was 2 significantly differed between males and females, before we conclude that male rats were more risk seeking than females, we have to exclude an alternative explanation. That is, the difference in body weight between the two sexes might account for the difference in choice rate. In this task, both males and females were given the same volume of water for each reward. Since females are lighter than males, even the same reward might have had a higher value for females; e.g., possibly two drops water was enough to satisfy light females but not enough for heavy males. That might be why males needed to take the risky choice. To test this possibility, we analyzed the correlation between choice rates when *x* was two and body weight in each sex group. However, there was no significant correlation (males: correlation coefficient = −0.25, *p* = 0.46, females: correlation coefficient = −0.18, *p* = 0.55 by Pearson product-moment correlation coefficient). Thus, we concluded that the difference in choice rate between males and females originated from a sex difference in risk preference.

### Experiment 2

The risky choice resulted in being either rewarded (win) or unrewarded (lose). Do males and females change their decision depending on a win/lose experience in the past trial? To address this question, we tested their performances in the choice of 4/0 drops vs. 2 drops and analyzed their choice history. In Experiment 2, male rats again exhibited higher risk seeking attitudes than females (the left-side bars in Figure 3B; *t*_(17)_ = 2.12, *p* = 0.04 by Welch’s* t*-test). The trials were then sorted according to the choice and outcome of the previous trial. The right-side bars of Figure 3B show the percent choices of risky option in the trial after winning (losing) and also that in the trial after two and three consecutive wins (loses). Significant sex difference was not observed across after winning(s) (Sex: *p* = 0.98 by two-way ANOVA [Sex × Number of win], Number of win: *p* = 0.04, interaction: *p* = 0.50) but was observed across after losing(s) (Sex: *p* = 0.04 by two-way ANOVA [Sex × Number of lose], Number of lose: *p* = 0.04, interaction: *p* = 0.59). The difference in choice preference between after winning(s) and losing(s) in females was prominent between one win and one lose. There, the choice rate was significantly lower after losing than winning in females (*t*_(12)_ = 2.99, *p* = 0.01 by paired *t*-test) whereas that was not in males (*t*_(10)_ = −0.22, *p* = 0.83 by paired *t*-test). The outcome history analysis revealed that sex different risk preference emerged in choices after losing (the choice performance data with each outcome sequence in the past two and three consecutive risky choices are shown in Table S1 in Supplementary Material).

**Figure 4:**
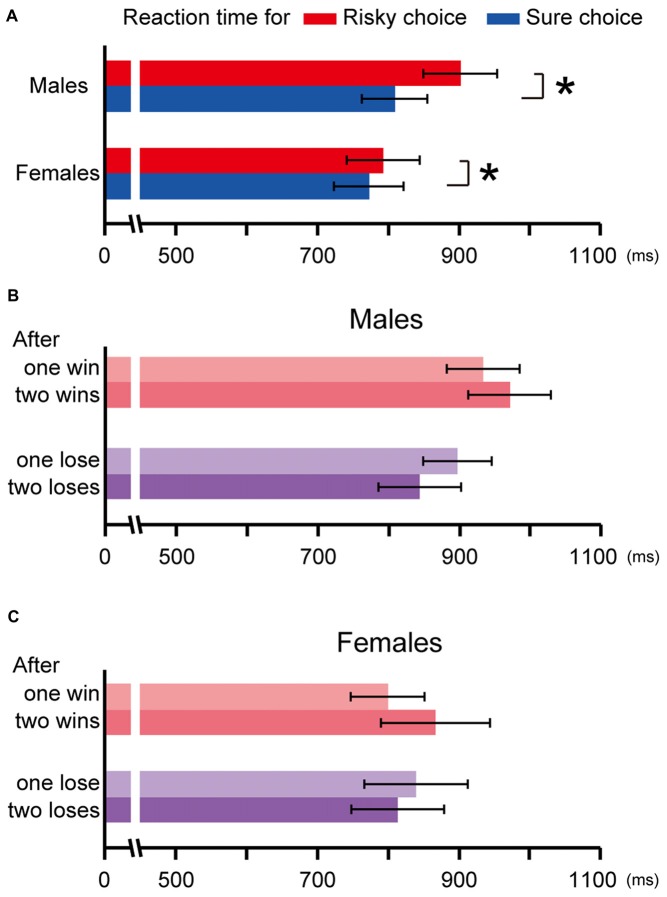
The reaction time in the risky choice trials and in the sure choice trials in Experiment 2 **(A)**. Both males and females spent more time choosing the risky option than choosing the sure option (*p* ≪ 0.01, *p* = 0.01, respectively, indicated by asterisks). The choice history analysis for the reaction time in males **(B)** and females **(C)**. The reaction time for the risky choice after winning was longer than that after losing in males (one win vs. lose: *p* = 0.07, two consecutive wins vs. loses: *p* ≪ 0.01), but was not significant in females (one win vs. lose: *p* = 0.56, two consecutive wins vs. loses: *p* = 0.26). Error bars indicate SEM.

We also analyzed the reaction time from gate open to the nose-poke response for making a choice. Since both males and females exhibited a preference for the risky option (males: *t*_(10)_ = 5.99, *p* ≪ 0.01, females: *t*_(12)_ = 5.66, *p* ≪ 0.01 by one sample *t*-test against chance level), the reaction time for the risky choice was expected to be the faster than that for the sure choice. However, the result was opposite; the reaction time for the risky choice was the slower than that for the sure choice in both sexes (males: *t*_(10)_ = 5.29, *p* ≪ 0.01, females: *t*_(12)_ = 2.89, *p* = 0.01 by paired *t*-test; Figure 4A). To test the sex difference, the ratios of the reaction time in the risky choice against those in the sure choice were compared because the reaction times widely differed among individuals and a comparison in terms of the actual value was thought to be not appropriate. The ratio was significantly larger in males than in females (*t*_(21)_ = 2.76, *p* = 0.01 by Welch’s *t-test*), which indicates that males spent much more time choosing the risky option than females did. In addition, the choice history analysis revealed the reaction time for the risky choice in males got longer as wins kept on, while it got shorter as losses kept on (Figure 4B). The reaction time for the risky choice in the trials after winning was the longer than that after losing (one win vs. lose: *t*_(10)_ = 1.99, *p* = 0.07, two consecutive wins vs. loses:* t*_(10)_ = 3.91, *p* < 0.01 by paired *t-test*). This tendency was not observed in females (one win vs. lose: *t*_(12)_ = −0.60, *p* = 0.56, two consecutive wins vs. loses: *t*_(12)_ = 1.20, *p* = 0.26 by paired *t*-test; Figure 4C).

### Experiment 3

**Figure 5:**
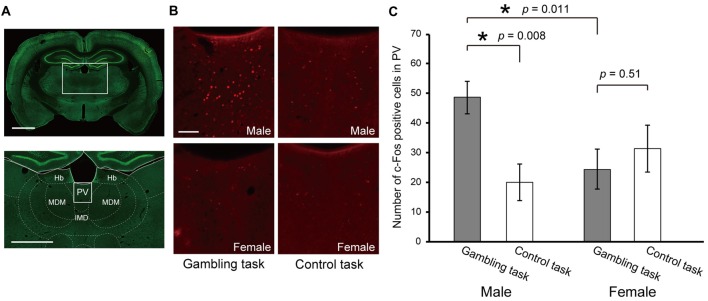
c-Fos expression in the PV after performance of the gambling task and control discrimination choice task. **(A,B)** Representative images of immunohistochemistry. NeuN positive cells are colored green (**A**, scale bars indicate 1000 μm) and c-Fos positive cells are colored red (**B**, scale bar indicates 100 μm). **(C)** Number of c-Fos positive cells in the PV. A comparison was conducted by two-way ANOVA [sex (male/female) × task type (gambling task/control task)]. The *p* values in the figure are from the *post hoc* simple main effect test. The significant *p* values are indicated by asterisks. Error bars indicate SEM. Abbreviations: Hb, habenular nucleus; PV, paraventricular thalamic nucleus; MDM, mediodorsal thalamic nucleus; IMD, intermediodorsal thalamic nucleus.

Lastly, to investigate the sex difference in neural activity during the performance of the gambling task, we conducted c-Fos immunohistochemistry. After the tests in Experiment 2, one subset of the rats (six males and six females) was subjected to a single session of the gambling task in the form of Experiment 1. As a control, the other subset (four males and three females) was subjected to a single session of a simple reward amount discrimination choice of 4 drops vs. 2 drops. The brains of both subsets were fixed 90 min after the behavioral tests. In this report, we focused on the paraventricular nucleus of the thalamus (PV), which is implicated in approaching/avoidance switching under unpredictable reward omission (Do-Monte et al., 2017) so that the PV is hypothesized to play a prominent role in gambling. Figure 5 shows the average number of c-Fos positive cells in the PV for each sex and task type. A two-way ANOVA [sex × task type] revealed a non-significant effect on sex (*F*_(1,15)_ = 2.52, *p* = 0.13) and task type (*F*_(1,15)_ = 3.29, *p* = 0.08), but a significant effect on the interaction (*F*_(1,15)_ = 6.64, *p* = 0.02). The following *post hoc* simple main effect test revealed a significant effect on task type in males (*F*_(1,8)_ = 9.47, *p* < 0.01), but did not in females (*F*_(1,7)_ = 0.46, *p* = 0.51). In addition, it revealed a significant effect on sex in the gambling task (*F*_(1,10)_ = 8.52, *p* = 0.01), but did not reveal any in the discrimination choice task (*F*_(1,5)_ = 1.05, *p* = 0.32). In summary, the PV showed preferentially high activity for the performance of gambling in males.

## Discussion

### Sex Difference in Risk Preference

In the present study, both male and female rats preferred the risky option when the risky and sure options had the same expected value. However, males chose the risky option much more frequently than females. That was replicated in Experiment 1 and 2. Importantly, when the sure option provided the best offer (*x* was 4), both males and females correctly chose the sure option with the same level of accuracy. Thus, it is thought that the ability to perform this choice task did not differ between the two sexes. Furthermore, sex difference in body weight cannot account for the sex difference in risky choice since the frequency of risky choices was not correlated with individual body weight. From these observations, we conclude that male rats were more risk seeking than females. However, note that individual differences also emerged within both sex groups.

In the present study, we did not refer to estrous cycle effects because any significant cyclic fluctuation in choice performance across estrous cycle was not found in our preliminary experiment. The previous studies also reported no estrous cycle effect on choice performance in the rIGT task (Georgiou et al., 2018) and in the risky decision making task with probabilistic foot-shock (Orsini et al., 2016). Changes in c-fos expression level in the PV across estrous cycle was also not found in the elevated plus maze task (Sayin et al., 2014). Thus, it is less likely that estrous cycle effected our results.

### Sex Different Features Emerged After Losing

Outcome-history analysis revealed sex difference in their choice pattern; females reduced risk preference after losing compared to after winning. One simple expectation of outcome-based choice behavior is that the rats keep choosing the risky option while it provides a reward but switch to the sure option when the risky option goes unrewarded, which is called “win-stay/lose-shift” behavior. The choice pattern in females might be matched to this behavioral pattern. In contrast, male rats showed the same level of risk preference after losing as after winning. However, this does not mean that the males did not care about the outcome of the previous trial, because the outcome-history effect was canceled out by the opposite traits between individuals. Two risk seeking rats out of 11 exhibited significantly higher risk preference after winning than after losing (win-stay), whereas the other two risk seeking rats exhibited significantly higher risk preference after losing (lose-stay). There was no individual female who exhibited lose-stay behavior whereas 4 out of 13 exhibited win-stay behavior. Thus, one characteristics in males against females was individuals who took lose-stay behavior.

The results of the reaction time analysis were surprising for us. Since both males and females preferred the risky choice, we expected that the reaction time for the risky choice would be faster than that for the sure choice. Or, since this task was free choice and the chosen option should have a higher value at each trial level, it is reasonable that the reaction time should be the same between risky and sure choice. However, the result was the third alternative; they spent more time making the risky choice than making the sure one. They seemed to hesitate in choosing the risky option although they preferred it. In addition, the choice history analysis revealed that the reaction time for the risky choice in males got shorter as losses kept on whereas that in females did not. This tendency in males was prominent in the two individuals who showed lose-stay choice behavior and was not in the two who showed win-stay choice behavior. However, this relationship should be tested in a future study because we did not have enough samples to conduct a statistical test in the present study.

Given that significant sex different features mainly emerged after losing, male and female rats may evaluate an unsuccessful outcome of their decision in different view. Female rats might be more sensitive to negative outcomes compared to males. In addition, we also put general consideration about behavior of rats in the present gambling task. As stated above, the relationship between risk preference and reaction time in the present task looks ambivalent. In addition, the risk preference and the reaction time dynamically changed on the micro scale, depending on the past win/lose experience. We think that these observations are clear evidence that their behaviors were not stereotypical but rather risky decision making.

### c-Fos Expression in PV

To the best of our knowledge, this is the first study reporting a sex difference in c-Fos expression for risky decision making. Among the brain regions, we focused on the paraventricular nucleus of the thalamus (PV). The PV is currently considered a key region that controls approaching/avoidance behavior switching, especially in lines of regulation of emotional response (Hsu et al., 2014; Kirouac, 2015; Choi and McNally, 2017). Recently, Do-Monte et al. (2017) found that the PV modulated the expression level of exploratory foraging behavior when the reward was unpredictably omitted and that the PV control was not recruited when the outcome was predicted. In the present gambling task as well, the outcome of the risky choice was unpredictable and resulted in reward omission in half of the trials. That means the present finding that c-Fos expression in male’s PV was higher for the gambling choice than for the stereotypic discrimination choice is consistent with the previous findings. In addition, c-Fos expression of the PV in the gambling task was higher in males than in females. This result implies that the PV is involved in the sex difference in risk preference and that might reflect a gap in sensitivity to negative outcomes between males and females. However, the present result is just correlation. A future study is needed to prove causal contribution of the PV to risk seeking behavior, where a possibility that the PV may respectively control approaching and avoidance via projections to the nucleus accumbens and amygdala (Vertes and Hoover, 2008) should be taken into account. In addition to the PV, the dopamine and noradrenaline systems which is thought to be associated with frustration and stress are other possible candidates responsible for sex different preference after losing.

### Why Did the Rats Choose the Risky Option More Frequently?

Finally, let us discuss the origin of their risk preference. To date, many animal experiments have reported risk seeking behavior in monkeys (Hayden and Platt, 2007; Hayden et al., 2008; Long et al., 2009; Watson et al., 2009; O’Neill and Schultz, 2010; So and Stuphorn, 2010; Heilbronner et al., 2011; Raghuraman and Padoa-Schioppa, 2014) and rats (Roitman and Roitman, 2010; Ishii et al., 2012, 2015). Just as we do not feel double value for a double plate of dinner, the subjective utility for reward amount is thought to be rarely linear, rather, concave-down like in the reward gain frame. Accordingly, it is predicted that the average utility of the double reward and no reward of a risky option is lower than the middle reward of a sure option, so that the subject should choose the sure one. Indeed, human behavior well matches this risk averse prediction (Tversky and Kahneman, 1981). However, recent study found that monkey’s gambling behavior could be described by a sigmoid utility function (Genest et al., 2016). Why did the experimental animals often prefer risky choices even though the risky and sure options were equal in total? They might behave with a short-term perspective and their utility reference might dynamically fluctuate trial by trial because they cared about single win/lose experiences. An alternative possibility is that repeated trials allowed them to think that they could make back the loss quickly in the following trials. Indeed, in the present study, the male rats did not decrease their risk preference after losing and exhibited a shorter reaction time for the risky choice after losing. The frequency of trials (interval time) is also thought to be an important factor (Heilbronner and Hayden, 2013). A longer inter-trial interval decreased risk preference (Hayden and Platt, 2007). Numerous trials and voluntary intervals might diminish their anxiety about a loss. However, this issue is open question and the other potent theory has been proposed (see Adriani and Laviola, 2016).

## Conclusion

We addressed the sex difference in risk preference of rats by using a new gambling task. We found that male rats were more risk seeking than females, which especially emerged in choices made after a losing gamble. In addition, c-Fos analysis found preferential high activity in the PV to male’s gambling. This finding opens a new idea that the PV is one candidate responsible for sex different risky decision making.

## Author Contributions

HI planned the design of the research. HI, MO and SO performed the experiments and analyzed the data. HI, SO, K-IT and TI wrote the article.

## Conflict of Interest Statement

The authors declare that the research was conducted in the absence of any commercial or financial relationships that could be construed as a potential conflict of interest.

## References

[B1] Adriani W. Laviola G. (2016). Commentary on the special issue “The Adolescent Brain”: how can we run operant paradigms in a preclinical adolescent model? Technical tips and future perspectives. Neurosci. Biobehav. Rev. 70, 323–328. 10.1016/j.neubiorev.2016.07.028 27484871

[B2] Arora M. Kumari S. (2015). Risk taking in financial decisions as a function of age, gender: mediating role of loss aversion and regret. Int. J. Appl. Psychol. 5, 83–89.

[B3] Boinski S. (1988). Sex differences in the foraging behavior of squirrel monkeys in a seasonal habitat. Behav. Ecol. Sociobiol. 23, 177–186. 10.1007/bf00300352

[B4] Burke C. J. Tobler P. N. (2011). Coding of reward probability and risk by single neurons in animals. Front. Neurosci. 5:121. 10.3389/fnins.2011.00121 22013410 PMC3190139

[B5] Caraco T. Martindale S. Whittam T. S. (1980). An empirical demonstration of risk-sensitive foraging preferences. Anim. Behav. 28, 820–830. 10.1016/s0003-3472(80)80142-4

[B6] Charness G. Gneezy U. (2012). Strong evidence for gender differences in risk-taking. J. Econ. Behav. Organ. 83, 50–58. 10.1016/j.jebo.2011.06.007

[B7] Choi E. A. McNally G. P. (2017). Paraventricular thalamus balances danger and reward. J. Neurosci. 37, 3018–3029. 10.1523/JNEUROSCI.3320-16.2017 28193686 PMC6596734

[B8] Clarke J. Manly B. Kerry K. Gardner H. Franchi E. Corsolini S. (1998). Sex differences in Adélie penguin foraging strategies. Polar Biol. 20, 248–258. 10.1007/s003000050301

[B9] Dalla C. Shors T. J. (2009). Sex differences in learning processes of classical and operant conditioning. Physiol. Behav. 97, 229–238. 10.1016/j.physbeh.2009.02.035 19272397 PMC2699937

[B10] Dienstbier R. A. French J. A. Kamil A. C. Leger D. W. (2001). Evolutionary Psychology and Motivation. Lincoln, NE: University of Nebraska Press.

[B11] Do-Monte F. H. Minier-Toribio A. Quiñones-Laracuente K. Medina-Colón E. M. Quirk G. J. (2017). Thalamic regulation of sucrose seeking during unexpected reward omission. Neuron 94, 388–400. 10.1016/j.neuron.2017.03.036 28426970 PMC5484638

[B12] Do-Monte F. H. Quirk G. J. Li B. Penzo M. A. (2016). Retrieving fear memories, as time goes by… Mol. Psychiatry 21, 1027–1036. 10.1038/mp.2016.78 27217148 PMC4956525

[B13] Dwyer P. D. Gilkeson J. H. List J. A. (2002). Gender differences in revealed risk taking: evidence from mutual fund investors. Econ. Lett. 76, 151–158. 10.1016/s0165-1765(02)00045-9

[B14] Eckel C. C. Grossman P. J. (2002). Sex differences and statistical stereotyping in attitudes toward financial risk. Evol. Hum. Behav. 23, 281–295. 10.1016/s1090-5138(02)00097-1

[B15] Genest W. Stauffer W. R. Schultz W. (2016). Utility functions predict variance and skewness risk preferences in monkeys. Proc. Natl. Acad. Sci. U S A 113, 8402–8407. 10.1073/pnas.1602217113 27402743 PMC4968724

[B16] Georgiou P. Zanos P. Bhat S. Tracy J. K. Merchenthaler I. J. McCarthy M. M.. (2018). Dopamine and stress system modulation of sex differences in decision making. Neuropsychopharmacology 43, 313–324. 10.1038/npp.2017.161 28741626 PMC5729565

[B17] Ginnett T. F. Demment M. W. (1997). Sex differences in giraffe foraging behavior at two spatial scales. Oecologia 110, 291–300. 10.1007/s004420050162 28307437

[B18] Glimcher P. W. Camerer C. F. Fehr E. Poldrack R. A. (2009). Neuroeconomics: Decision Making and the Brain. London: Academic Press.

[B19] Hammerslag L. R. Gulley J. M. (2014). Age and sex differences in reward behavior in adolescent and adult rats. Dev. Psychobiol. 56, 611–621. 10.1002/dev.21127 23754712 PMC4782597

[B20] Hayden B. Y. Heilbronner S. R. Nair A. C. Platt M. L. (2008). Cognitive influences on risk-seeking by rhesus macaques. Judgm. Decis. Mak. 3, 389–395. 19844596 PMC2763334

[B21] Hayden B. Y. Platt M. L. (2007). Temporal discounting predicts risk sensitivity in rhesus macaques. Curr. Biol. 17, 49–53. 10.1016/j.cub.2006.10.055 17208186 PMC1868415

[B22] Heilbronner S. R. Hayden B. Y. (2013). Contextual factors explain risk-seeking preferences in rhesus monkeys. Front. Neurosci. 7:7. 10.3389/fnins.2013.00007 23378827 PMC3561601

[B23] Heilbronner S. R. Hayden B. Y. Platt M. L. (2011). Decision salience signals in posterior cingulate cortex. Front Neurosci 5:55. 10.3389/fnins.2011.00055 21541308 PMC3082768

[B24] Hsu D. T. Kirouac G. J. Zubieta J. K. Bhatnagar S. (2014). Contributions of the paraventricular thalamic nucleus in the regulation of stress, motivation and mood. Front. Behav. Neurosci. 8:73. 10.3389/fnbeh.2014.00073 24653686 PMC3949320

[B25] Ishii H. Ohara S. Tobler P. N. Tsutsui K. I. Iijima T. (2012). Inactivating anterior insular cortex reduces risk taking. J. Neurosci. 32, 16031–16039. 10.1523/JNEUROSCI.2278-12.2012 23136439 PMC6621622

[B26] Ishii H. Ohara S. Tobler P. N. Tsutsui K. I. Iijima T. (2015). Dopaminergic and serotonergic modulation of anterior insular and orbitofrontal cortex function in risky decision making. Neurosci. Res. 92, 53–61. 10.1016/j.neures.2014.11.009 25481848

[B27] Kirouac G. J. (2015). Placing the paraventricular nucleus of the thalamus within the brain circuits that control behavior. Neurosci. Biobehav. Rev. 56, 315–329. 10.1016/j.neubiorev.2015.08.005 26255593

[B28] Le Boeuf B. J. Crocker D. E. Blackwell S. B. Morris P. A. Thorson P. H. (1993). Sex differences in diving and foraging behaviour of northern elephant seals. Symp. Zool. Soc. Lond. 66, 149–178.

[B29] Logan F. A. (1965). Decision making by rats: uncertain outcome choices. J. Comp. Physiol. Psychol. 59, 246–251. 10.1037/h0021850 14288349

[B30] Long A. B. Kuhn C. M. Platt M. L. (2009). Serotonin shapes risky decision making in monkeys. Soc. Cogn. Affect. Neurosci. 4, 346–356. 10.1093/scan/nsp020 19553236 PMC2799948

[B31] O’Neill M. Schultz W. (2010). Coding of reward risk by orbitofrontal neurons is mostly distinct from coding of reward value. Neuron 68, 789–800. 10.1016/j.neuron.2010.09.031 21092866

[B32] Orsini C. A. Willis M. L. Gilbert R. J. Bizon J. L. Setlow B. (2016). Sex differences in a rat model of risky decision making. Behav. Neurosci. 130, 50–61. 10.1037/bne0000111 26653713 PMC4738105

[B33] Paxinos G. Watson C. (2007). The Rat Brain in Stereotaxic Coordinates. 6th Edn. London: Academic.

[B34] Peak J. N. Turner K. M. Burne T. H. J. (2015). The effect of developmental vitamin D deficiency in male and female Sprague-Dawley rats on decision-making using a rodent gambling task. Physiol. Behav. 138, 319–324. 10.1016/j.physbeh.2014.09.007 25447469

[B35] Platt M. L. Huettel S. A. (2008). Risky business: the neuroeconomics of decision making under uncertainty. Nat. Neurosci. 11, 398–403. 10.1038/nn2062 18368046 PMC3065064

[B36] Powell M. Ansic D. (1997). Gender differences in risk behaviour in financial decision-making: An experimental analysis. J. Econ. Psychol. 18, 605–628. 10.1016/s0167-4870(97)00026-3

[B37] Raghuraman A. P. Padoa-Schioppa C. (2014). Integration of multiple determinants in the neuronal computation of economic values. J. Neurosci. 34, 11583–11603. 10.1523/jneurosci.1235-14.2014 25164656 PMC6608411

[B38] Raylu N. Oei T. P. (2002). Pathological gambling: a comprehensive review. Clin. Psychol. Rev. 22, 1009–1061. 10.1016/S0272-7358(02)00101-0 12238245

[B39] Roitman J. D. Roitman M. F. (2010). Risk-preference differentiates orbitofrontal cortex responses to freely chosen reward outcomes. Eur. J. Neurosci. 31, 1492–1500. 10.1111/j.1460-9568.2010.07169.x 20384776 PMC3745026

[B40] Sayin A. Derinöz O. Yüksel N. Şahin S. Bolay H. (2014). The effects of the estrus cycle and citalopram on anxiety-like behaviors and c-fos expression in rats. Pharmacol. Biochem. Behav. 124, 180–187. 10.1016/j.pbb.2014.06.002 24933337

[B41] So N. Y. Stuphorn V. (2010). Supplementary eye field encodes option and action value for saccades with variable reward. J. Neurophysiol. 104, 2634–2653. 10.1152/jn.00430.2010 20739596 PMC4073903

[B42] St. Onge J. R. Chiu Y. C. Floresco S. B. (2010). Differential effects of dopaminergic manipulations on risky choice. Psychopharmacology 211, 209–221. 10.1007/s00213-010-1883-y 20495787

[B43] Stephens D. W. Krebs J. R. (1986). Foraging Theory. Princeton: Princeton University Press.

[B44] Tversky A. Kahneman D. (1981). The framing of decisions and the psychology of choice. Science 211, 453–458. 10.1126/science.7455683 7455683

[B45] van den Bos R. Davies W. Dellu-Hagedorn F. Goudriaan A. E. Granon S. Homberg J.. (2013a). Cross-species approaches to pathological gambling: a review targeting sex differences, adolescent vulnerability and ecological validity of research tools. Neurosci. Biobehav. Rev. 37, 2454–2471. 10.1016/j.neubiorev.2013.07.005 23867802

[B46] van den Bos R. Homberg J. de Visser L. (2013b). A critical review of sex differences in decision-making tasks: focus on the Iowa Gambling Task. Behav. Brain Res. 238, 95–108. 10.1016/j.bbr.2012.10.002 23078950

[B47] van den Bos R. Jolles J. van der Knaap L. Baars A. de Visser L. (2012). Male and female Wistar rats differ in decision-making performance in a rodent version of the Iowa Gambling Task. Behav. Brain Res. 234, 375–379. 10.1016/j.bbr.2012.07.015 22814113

[B48] Vertes R. P. Hoover W. B. (2008). Projections of the paraventricular and paratenial nuclei of the dorsal midline thalamus in the rat. J. Comp. Neurol. 508, 212–237. 10.1002/cne.21679 18311787

[B49] Watson K. K. Ghodasra J. H. Platt M. L. (2009). Serotonin transporter genotype modulates social reward and punishment in *rhesus macaques*. PLoS One 4:e4156. 10.1371/journal.pone.0004156 19142220 PMC2612746

[B50] Weber E. U. Blais A. R. Betz N. E. (2002). A domain-specific risk-attitude scale: measuring risk perceptions and risk behaviors. J. Behav. Decis. Mak. 15, 263–290. 10.1002/bdm.414

[B51] Williams G. C. (1957). Pleiotropy, natural selection, and the evolution of senescence. Evolution 11, 398–411. 10.2307/2406060

[B52] Zoratto F. Laviola G. Adriani W. (2016). The subjective value of probabilistic outcomes: impact of reward magnitude on choice with uncertain rewards in rats. Neurosci. Lett. 617, 225–231. 10.1016/j.neulet.2016.02.026 26905669

