# Supplementary material for: Sex Differences in Risk Preference and c-Fos Expression in Paraventricular Thalamic Nucleus of Rats During Gambling Task
Source: Front Behav Neurosci. 2018 Apr 10;12:68. doi: 10.3389/fnbeh.2018.00068 (PMC5902494; doi:10.3389/fnbeh.2018.00068)
Supplement: Supplementary file 1 [file Table_1.DOCX]

***Supplementary Material***

**Sex differences in risk preference and c-Fos expression in** **paraventricular thalamic nucleus of rats during gambling task**

**Hironori Ishii, Mariko Onodera, Shinya Ohara, Ken-Ichiro Tsutsui, Toshio Iijima**

*** Correspondence: Hironori Ishii: hironoriishii141@gmail.com**

**Supplementary Table 1.** Choice performance and outcome sequence in past 2 and 3 consecutive risky choice trials. The values in males and females indicate mean ± sem (%). of the percent choice of the risky option. The p values are the results of comparison in the percent choice of the risky option between males and females by t-test (significance level: p < 0.05). The t-1, 2, 3 indicate the outcome of risky choice in the past 1, 2, 3 trial. “W” indicates win, “L” indicates lose.

| 2 trial back | |  |  |  |  |  |  |  |
| --- | --- | --- | --- | --- | --- | --- | --- | --- |
| males | 62.9±2.7 | 61.9±3.2 | 63.2±4.4 | 62.1±3.6 |  |  |  |  |
| females | 63.3±4.8 | 56.8±3.4 | 49.3±3.1 | 55.3±3.9 |  |  |  |  |
| p value | 0.95 | 0.3 | 0.018 | 0.21 |  |  |  |  |
| t-1 | W | W | L | L |  |  |  |  |
| t-2 | W | L | W | L |  |  |  |  |
|  |  |  |  |  |  |  |  |  |
| 3 trial back | |  |  |  |  |  |  |  |
| males | 65.6±5.1 | 64.7±3.2 | 70.7±4.9 | 62.8±4.5 | 60.4±5.1 | 68.6±5.5 | 63.1±5.4 | 67.9±5.4 |
| females | 68.3±5.3 | 66.4±5.6 | 60.1±4.4 | 61.5±3.6 | 53.8±5 | 53.3±3.1 | 57.4±4.4 | 61.8±7 |
| p value | 0.71 | 0.8 | 0.12 | 0.82 | 0.37 | 0.03 | 0.42 | 0.5 |
| t-1 | W | W | W | W | L | L | L | L |
| t-2 | W | W | L | L | W | W | L | L |
| t-3 | W | L | W | L | W | L | W | L |
